# Epidemiology and pathogenicity of *Haemophilus* parasuis in eastern China

**DOI:** 10.3389/fmicb.2025.1589975

**Published:** 2025-05-15

**Authors:** Jingyu Xu, Xin Jin, Xuesong Li, Dehong Yang

**Affiliations:** ^1^College of Veterinary Medicine, South China Agricultural University, Guangzhou, China; ^2^Guangdong Enterprise Key Laboratory for Animal Health and Environmental Control, Wen's Foodstuff Group Co. Ltd., Yunfu, China

**Keywords:** *Haemophilus parasuis*, epidemiology, serotype, virulence gene, pathogenicity

## Abstract

*Haemophilus parasuis* (*H. parasuis*), the causative agent of Glässer’s disease (polyserositis), exhibits considerable serotype diversity and is globally distributed. To investigate the epidemiological characteristics of *H. parasuis* in China, 810 suspected infection samples, including lung tissues and pleural effusions, were systematically analyzed. These samples were collected between 2022 and 2024 from six major pig-producing provinces in China. The analysis revealed a national infection rate of 52.10% (422/810), with Jiangxi Province exhibiting the highest prevalence (71.76%). Seasonal analysis indicated significantly higher incidence rates during winter (66.29%) and spring (60.45%) compared to summer (34.81%) and autumn (46.46%). Serotyping of 56 *H. parasuis* isolates demonstrated that serotype 5 (42.86%) and serotype 12 (19.64%) were predominant, while 10.71% of the strains were nontypeable. Notably, Jiangxi Province displayed a single-serotype profile, whereas other provinces exhibited multiple-serotype cocirculation patterns. Virulence gene analysis revealed the universal absence of *HPM-1370* and the consistent presence of *vta3* across all isolates. Serotypes 4 and 8 exclusively harbored the *vta* gene cluster, while serotype 5 retained other target genes despite lacking *HPM-1370*. The *HPM-1371* gene was detected only in serotypes 5 and 14. Serotypes 1, 11, 12, and 13 exhibited a combination of conserved *wza-vta1-vta2 -vta3* genes, although 36.37% (4/11) of serotype 12 isolates lacked the *wza* gene. Serotypes 2 and 7 carried only *wza* and *vta3* genes. Animal challenge experiments demonstrated marked differences in strain pathogenicity: the H5-1 strain induced 100% mortality with acute septicemia, widespread alveolar destruction, and fibrinous exudation; the H12-1 strain caused 50% mortality accompanied by severe pleural adhesions and hemorrhagic lesions; and the H7-1 strain resulted in 33.33% mortality with localized pulmonary damage. These findings provide essential evidence for the development of targeted prevention and control strategies against *H. parasuis* infection.

## 1 Introduction

*H. parasuis*, a member of the Pasteurellaceae family and belonging to the genus *Haemophilus*, is a gram-negative, pleomorphic coccobacillus that typically colonizes the upper respiratory mucosa of swine (He et al., 2018). The disease caused by *H. parasuis* is characterized by fibrinous polyserositis, arthritis, and meningitis, accompanied by severe inflammatory responses and tissue damage, *which* substantially *impair* swine health and production performance (Macedo et al., 2015).

The pathogenicity of *H. parasuis* is critically determined by its virulence factors. The *lsgB* gene encodes a sialyltransferase involved in lipooligosaccharide synthesis, facilitating immune evasion (Wang et al., 2018); *capD* governs capsular polysaccharide biosynthesis, providing protection against host immunity; and *wza* encodes a polysaccharide export protein essential for capsule integrity (Martinez-Moliner et al., 2012). Additionally, *HPM-1370*, *HPM-1371*, *HPM-1372*, and *HPM-1370* encode glycosyltransferases involved in polysaccharide modification, which potentially influence bacterial adhesion and immune escape (Lawrence et al., 2015). The *vta1*, *vta2*, and *vta3* genes encode virulence-associated autotransporters that mediate host-pathogen interactions, including adhesion, invasion, and immune evasion (Olvera et al., 2012). The synergistic action of these factors enables *H. parasuis* to effectively colonize hosts and exacerbate clinical disease, thereby complicating control measures.

*H. parasuis* is classified into 15 serotypes based on capsular polysaccharide gene variations, with additional nontypeable strains (Oliveira et al., 2003). These serotypes do not exhibit cross-immunity, demonstrate strong antimicrobial resistance, and differ markedly in clinical manifestations, pathogenicity, and host immune responses (Yang et al., 2020). Serotypes 1, 5, 12, 13, and 14 are regarded as highly virulent and are associated with high mortality rates (Kielstein and Rapp-Gabrielson, 1992); serotypes 2, 4, 8, and 15 exhibit moderate virulence and typically cause septicemia (Nielsen, 1993); while serotypes 3, 6, 7, 9, and 11 show low virulence and are often associated with asymptomatic infection. Serotypes 4, 5, 12, 13, and 14 predominate in China (Duan et al., 2025). Notably, changes in swine farming practices and the emergence of immunosuppressive viruses have increased the incidence of *H. parasuis* coinfections with other pathogens (e.g., bacteria and mycoplasma), leading to higher disease prevalence and causing substantial global economic losses.

In recent years, *H. parasuis* has caused severe economic impacts *on* swine farms in China. To address this issue, a systematic study was conducted from February 2022 to December 2024, analyzing 810 suspected *H. parasuis*-infected samples, including lung tissues and anal swabs, from six provinces in China. This study aimed to provide a scientific basis for improving prevention and therapeutic strategies against *H. parasuis*.

## 2 Materials and methods

### 2.1 Ethical statement

Animal infection experiments were conducted in accordance with the Guidelines for Experimental Animals issued by the Ministry of Science and Technology of China (Beijing). They were approved by the National Committee for Animal Ethics and Utilization. Ethical approval was obtained from South China Agricultural University (Approval No.: SYXK-2019––0136).

### 2.2 Sample collection

From 2022 to 2024, a total of 810 samples were collected from suspected cases of *H. parasuis* disease in 6 major pig-producing provinces in China (Guangdong, Anhui, Jiangsu, Shaanxi, Shandong, and Jiangxi). The samples were sourced from over 65 pig farms (with a total of 2 million pigs) and encompassed: 198 nasopharyngeal swabs, 277 pleural effusion samples, and 355 lung tissue samples. Affected pigs mainly manifested fever, cough, emaciation, and anorexia. Post-mortem examinations disclosed polyserositis, including fibrinous pleurisy, pericarditis, and yellow effusion coating the surfaces of thoracic and abdominal organs. This dataset reflects the clinical and pathological characteristics of *H. parasuis* infections in modern intensive pig farming systems.

### 2.3 Bacterial isolation and identification

Bacterial isolation was performed according to previously described methods. Briefly, internal tissues were aseptically streaked onto tryptic soy agar (TSA; Difco, Detroit, MI, United States) supplemented with 5% fetal bovine serum (FBS; Solarbio, China) and 1 μg/mL NAD (Sigma, St. Louis, MO, United States). The plates were incubated at 37°C under 5% CO_2_ for 18–24 h. Single colonies were subcultured in tryptic soy broth supplemented with 5% FBS and 1 μg/mL NAD at 37°C for 12–16 h. Gram staining was performed using a Gram Stain Kit (Solarbio), and bacterial morphology was observed microscopically (Kielstein et al., 2001; Moller et al., 1993).

One to two suspected colonies were selected for confirmation by PCR. The bacterial suspensions were heated at 100°C for 5 min and centrifuged at 8000–9000 rpm for 5 min to collect genomic DNA from the supernatant. Specific primers reported in the literature were used for the PCR identification of *H. parasuis* (Oliveira et al., 2001). The primers were synthesized by Sangon Biotech (Shanghai) Co., Ltd. (Table 1). The PCR was performed in a 20 μL reaction system containing 10 μL of 2 × Taq Mix, 1 μL of each primer, 1 μL of DNA template, and 5 μL of ddH_2_O. The cycling conditions were as follows: 94°C for 3 min; 35 cycles of 94°C for 30 s, 55°C for 30 s, and 72°C for 1 min; followed by a final extension at 72°C for 10 min.

**Table 1:** Primers for identifying and serotyping of *Haemophilus parasuis* (Oliveira et al., 2001; Jia et al., 2017).

Primer name	Primer sequence (5′ ~ 3′)	Annealing temperature	Product size
Hps-F	GTG ATG AGG AAG GGT GGT GT	56°C	821 bp
Hps-R	GGC TTC GTC ACC CTC TGT	56°C	
Hps1-F	TGCATAAAAAATTTTTGAA	49°C	1,245 bp
Hps1-R	TTATATATATTTTACATTTCTAAG		
Hps2-F	ATGGAAGAAAAAGAATATATC	52°C	1,032 bp
Hps2-R	TTAAAGTTTTGATTTGTCAATG		
Hps3-F	ATGACTAAAAAAATTTTAGTTACAG	52°C	1,068 bp
Hps3-F	TTACTTAATACCTAAGCG		
Hps4-F	ATGAATAATAAAGTCTCAATTATAA	52°C	753 bp
Hps4-R	TTACATATGTTTTACAATTCC		
Hps5-F	ATGCCAATAGAGATAGC	52°C	560 bp
Hps5-R	CCTGCCATATTATGA		
Hps6-F	ATGAGTATTTTTTTTCTAATTG	52°C	443 bp
Hps6-R	TTCCCTGATCATTGTAGTAACC		
Hps7-F	TAGTTGGTATATTATTTTCT	52°C	600 bp
Hps7-R	AGAATGCATCTGTACCACTAAG		
Hps8-F	CAGCAGGTTCTATGGAGTCA	49°C	350 bp
Hps8-R	CACATTATAACTTTCTTT		
Hps9-F	GCTCCAATATCAGCAGTA	58°C	819 bp
Hps9-R	AGAGTAATGAGCATCTCCG		
Hps10-F	TGATTATTCTACTGCCTTTA	55°C	320 bp
Hps10-R	CACCTAGCGTAACCCATA		
Hps11-F	ATGATTATAGGTATTTATGGTGC	52°C	657 bp
Hps11-R	CTATTTATTTTTTGAAAATTCTC		
Hps12-F	ATGGCTCACGATCCGAAAG	60°C	508 bp
Hps12-R	ATTTCCCTTTCCTAAACGC		
Hps13-F	GGCATTAGAGTTTCACCTA	60°C	800 bp
Hps13-R	TATTAGCATACCCAGCAT		
Hps14-F	TGTCTTTGTTACTACTAATTATTG	51°C	906 bp
Hps14-R	TAGTAACTCCAGATAAAGC		
Hps15-F	TTCGCAAGTATAAGGGACT	62°C	536 bp
Hps15-R	GATGTAGCCATAAAGTCAAT		

### 2.4 Serotyping

Serotyping was performed as described previously (Table 1) (Jia et al., 2017). Bacterial DNA was extracted by heating at 100°C for 5 min and centrifugation at 8000–9000 rpm for 5 min. PCR amplification was conducted using 2 × Taq Quick-Load Master Mix (CW Biotech, Beijing, China) with primers listed in Table 1, synthesized by Sangon Biotech Co., Ltd. (Shanghai, China). The thermal cycling conditions were as follows: 95°C for 3 min; 34 cycles of 95°C for 30 s, 56°C for 60 s, and 72°C for 1 min; followed by a final extension at 72°C for 5 min. Triplicate analyses were performed, and the products were resolved by 2% agarose gel electrophoresis.

### 2.5 Virulence gene identification

The virulence genes of *H. parasuis* were identified following previously described methodologies (Lawrence et al., 2015). Target genes, including *lsgB*, *capD*, *wza*, *HPM-1370*, *HPM-1371*, *HPM-1372*, *vta1*, *vta2*, *and vta3*, were amplified through PCR using primers listed in Table 2, which were commercially synthesized by Sangon Biotech (Shanghai) Co., Ltd. (Table 1). Each PCR reaction was conducted in a 20 μL system containing 10 μL of 2 × Taq Mix, 1 μL of each primer, 1 μL of genomic DNA template, and 7 μL of ddH_2_O. Thermal cycling parameters consisted of an initial denaturation at 94°C for 3 min, followed by 35 cycles of denaturation at 94°C for 30 s, annealing at 55°C for 30 s, and extension at 72°C for 1 min, with a final extension at 72°C for 10 min. All reactions were performed in triplicate, and amplification products were subsequently subjected to electrophoretic analysis using 2% agarose gels.

**Table 2:** Primers for identifying virulence genes of *Haemophilus parasuis* (Lawrence et al., 2015).

Genes	Primer name	Primer sequence (5′ ~ 3′)	Annealing temperature	Product size
lsgB	lsgB-F969	ATGAATTTGATTATTTGTATGACTCCATTTC	55°C	969 bp
	lsgB-R969	CTATTGGCATGTGTAGTCAATTACTTC		
capD	capD-F780	ATGTTAATGCCATTAATTTATTCATTG	55°C	780 bp
	capD-R780	TCGAACCGATAGAACCAGCAGCACCAGTC		
wza	wza-F840	ATGTGTAAGTTAACTAAAGCTCTTG	55°C	840 bp
	wza-R840	AGCAATTGCTTCGGTTAACGTCATAC		
1,370	1,370-F540	ATGCTAAAAAGAGTGTTTGATATTTTC	55°C	540 bp
	1,370-R540	TATATTATGATTAACATAATC		
1,371	1,371-F520	ATGAACTTTCTACCATTCGCCCTTCCCG	55°C	520 bp
	1,371-R520	ATTATATTTGAATCCAGGTTCAATG		
1,372	1,372-F720	ATGAAATTGTCTGTCTTAATGGCTGT	55°C	720 bp
	1,372-R720	TCCGCCAAATGTACATCATCAC		
1,373	1,373-F462	ATGAAATTGTCTGTCTTAATGGCTGT	55°C	462 bp
	1,373-R462	CTCTCATACCATACCCCAACTCAGG		
vta1	vta1-F406	TTTAGGTAAAGATAAGCAAGGAAATCC	55°C	406 bp
	vta1-R406	CCACACAAAACCTACCCCTCCTCC		
vta2	vta2-F294	AGCTTATATTCTCAGCACAAGGTGC	55°C	294 bp
	vta2-R294	CCACTGATAACCTACCCCCACAGAG		
vta3	vta3-F293	AATGGTAGCCAGTTGTATAATGTTGC	55°C	293 bp
	vta3-R293	CCACTGTAATGCAATACCTGCACC		

### 2.6 Animal pathogenicity assay

To evaluate the pathogenicity of the dominant serotypes of *Haemophilus parasuis*, 24 healthy 42-day-old Landrace pigs were selected. All animals tested negative for *Haemophilus parasuis* and other exogenous pathogens, including Classical Swine Fever (CSF), African Swine Fever (ASF), Porcine Reproductive and Respiratory Syndrome (PRRS), and Porcine Circovirus (PCV), in both antigen and antibody screening. The pigs were subsequently randomized into four experimental groups (n = 6 per group): three challenge groups (H5-1 [serotype 5], H7-1 [serotype 7], and H12-1 [serotype 12]) and a control group. The challenge groups were intraperitoneally injected with 2 mL of bacterial suspension (1.0 × 10^6^ CFU/mL), whereas the control group received 2 mL of sterile PBS. Clinical outcomes were monitored for 7 days post-inoculation. Deceased piglets were subjected to necropsy for pathological examination, and lung and liver tissues were collected for hematoxylin and eosin (H&E) staining. All experimental procedures were conducted in accordance with the Guidelines for Experimental Animals, and surviving piglets were euthanized by intravenous sodium pentobarbital injection to ensure welfare compliance.

### 2.7 Data analysis

The analysis and chart drawing of the infection rate of *Haemophilus parasuis*, the identification analysis of serotypes, the association between serotypes and virulence genes, etc. are completed through Office 2021 software. The relevant analysis and chart drawing of the survival rate in animal experiments are completed using GraphPad Prism 8.

## 3 Results

### 3.1 Detection of *Haemophilus parasuis* from clinical samples

**Figure 1:**
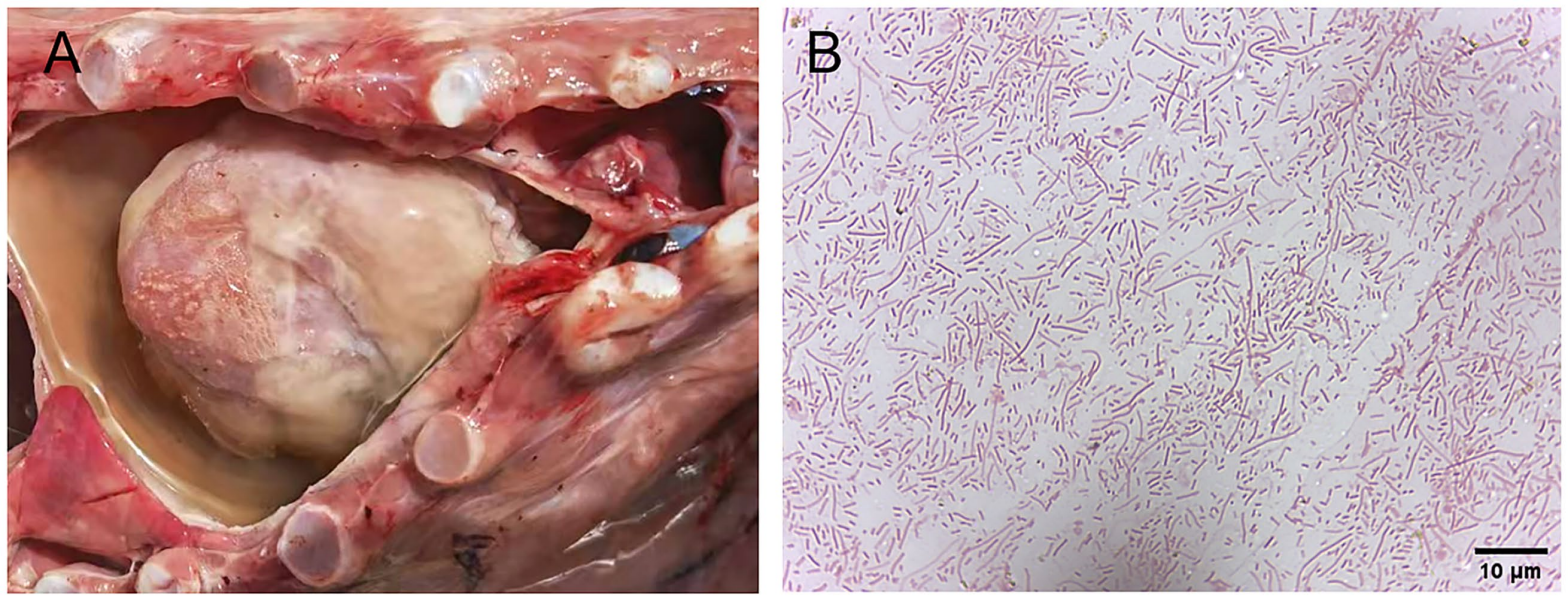
Gross lesions of pigs infected with *H. parasuis* and Gram staining of the suspected isolates of *H. parasuis*. **(A)** Fibrinous exudates and yellowish effusion are observed in the pericardium. **(B)** Gram staining of the suspected isolates of *H. parasuis*.

Field investigations identified typical symptoms of *H. parasuis* infection in affected swine herds, including pericarditis characterized by fibrinous adhesions between the pericardium and heart, forming a “shaggy heart” appearance, pleural fibrinous exudate, and fibrinous effusions in the thoracic cavities with pleural fluid accumulation (Figure 1A). The suspected *H. parasuis* strain, isolated from diseased materials, was cultured on tryptic soy agar and chocolate agar plates for 24 h. Gram staining and microscopic examination confirmed that the cultural characteristics and morphology of the isolated strain were consistent with those of the standard *H. parasuis* strain (Figure 1B).

To assess the infection status of *H. parasuis* in pigs across six provinces in China, PCR technology was employed to analyze 810 tissue samples suspected of *H. parasuis* infection, collected between 2022 and 2024. The results indicated an overall infection rate of 52.10% (422/810), with 56 strains of *H. parasuis* successfully isolated, corresponding to an isolation rate of 13.27% (56/422). Detailed information on these 56 isolated strains is presented in Supplementary Table 1.

**Figure 2:**
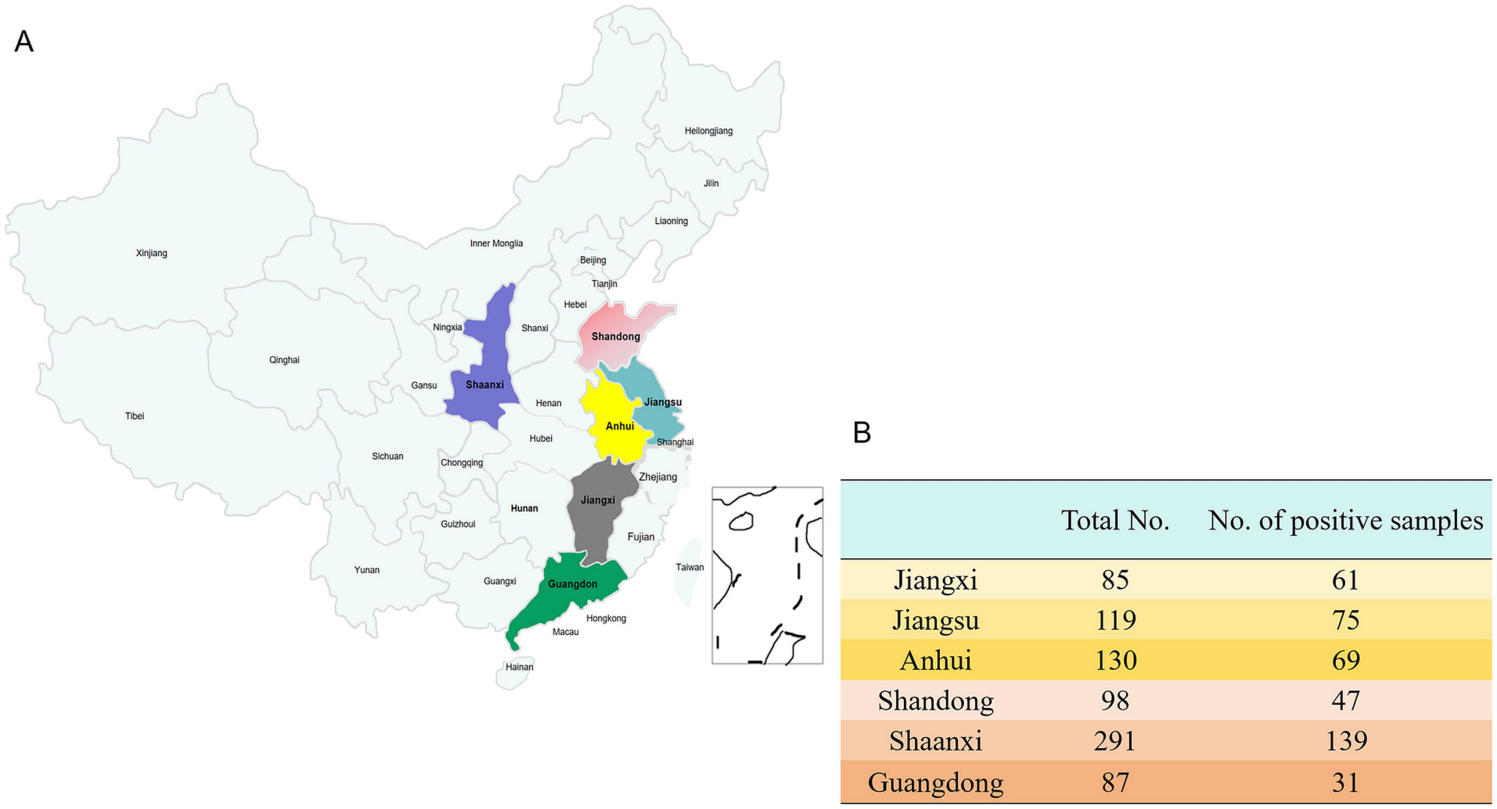
The prevalence of *H. parasuis* in more than 65 different pig farms across six regions of China. **(A)** The distribution of clinical samples from each pig farm. **(B)** The number of samples collected from six different regions in China and the number of samples with a positive result for *H. parasuis*.

**Figure 3:**
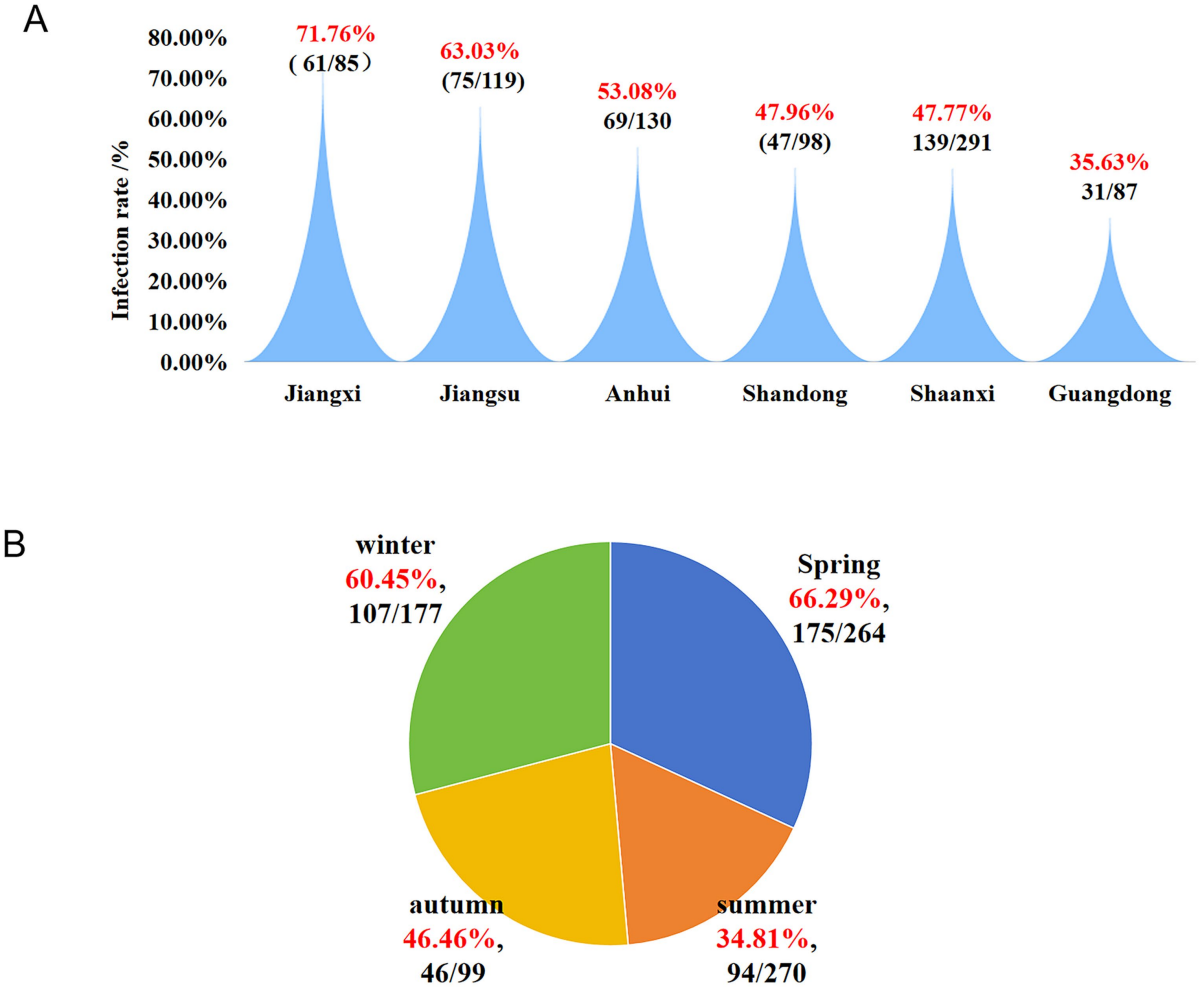
Infection status of *H. parasuis* in six different regions of China and its infection trends across the four seasons. **(A)** *H. parasuis* infection trends in various regions. **(B)** Infection trend of *H. parasuis* in different seasons.

Regarding provincial distribution (Figure 2), Jiangxi Province exhibited the highest infection rate at 71.76% (61/85), followed by Jiangsu Province at 63.03% (75/119) and Anhui Province at 53.08% (69/130). In contrast, the infection rates in Shandong Province (47.96%, 47/98), Shaanxi Province (47.77%, 139/291), and Guangdong Province (35.63%, 31/87) were comparatively lower (see Figure 3A).

To investigate the seasonal epidemic patterns of *H. parasuis*, a statistical analysis was conducted on samples collected between 2022 and 2024, categorized into the four seasons: spring, summer, autumn, and winter. The results indicated that the infection rate was highest in spring, reaching 66.29% (175/264), followed by winter at 60.45% (107/177). In contrast, the infection rates in summer and autumn were relatively lower, at 34.81% (94/270) and 46.46% (46/99), respectively (Figure 3B).

### 3.2 Serotype distribution and trends

**Figure 4:**
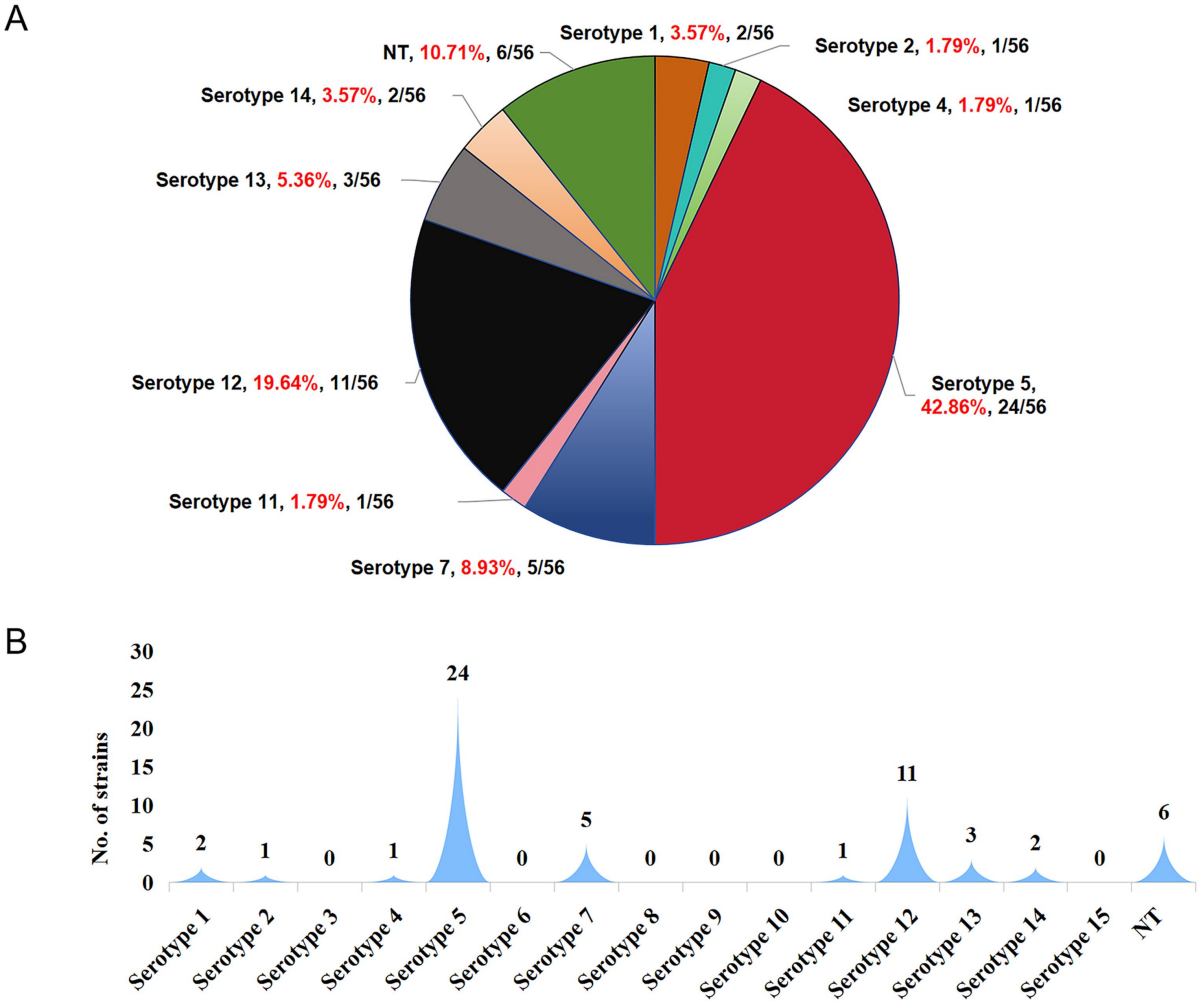
Distribution characteristics of serotypes of 56 *H. parasuis* isolates successfully obtained from *H. parasuis*-positive samples. **(A)** Prevalence percentage of each serotype. **(B)** The number of isolates corresponding to each serotype. NT represents nontypeable.

The prevalence of serotypes (Figure 4A) was as follows: serotype 5 exhibited the highest prevalence at 42.86% (24/56), followed by serotype 12 at 19.64% (11/56). Serotype 7 accounted for 8.93% (5/56), serotype 13 for 5.36% (3/56), while serotypes 1 and 14 each accounted for 3.57% (2/56). Serotypes 2, 4, and 11 each accounted for 1.79% (1/56). Additionally, 10.71% (6/56) of the isolates were non-typable. Serotypes 3, 6, 9, 10, and 15 were not detected in this study. Further analysis showed that among the 56 isolates, 50 were successfully typed, while the remaining six could not be classified (Figure 4B).

**Figure 5:**
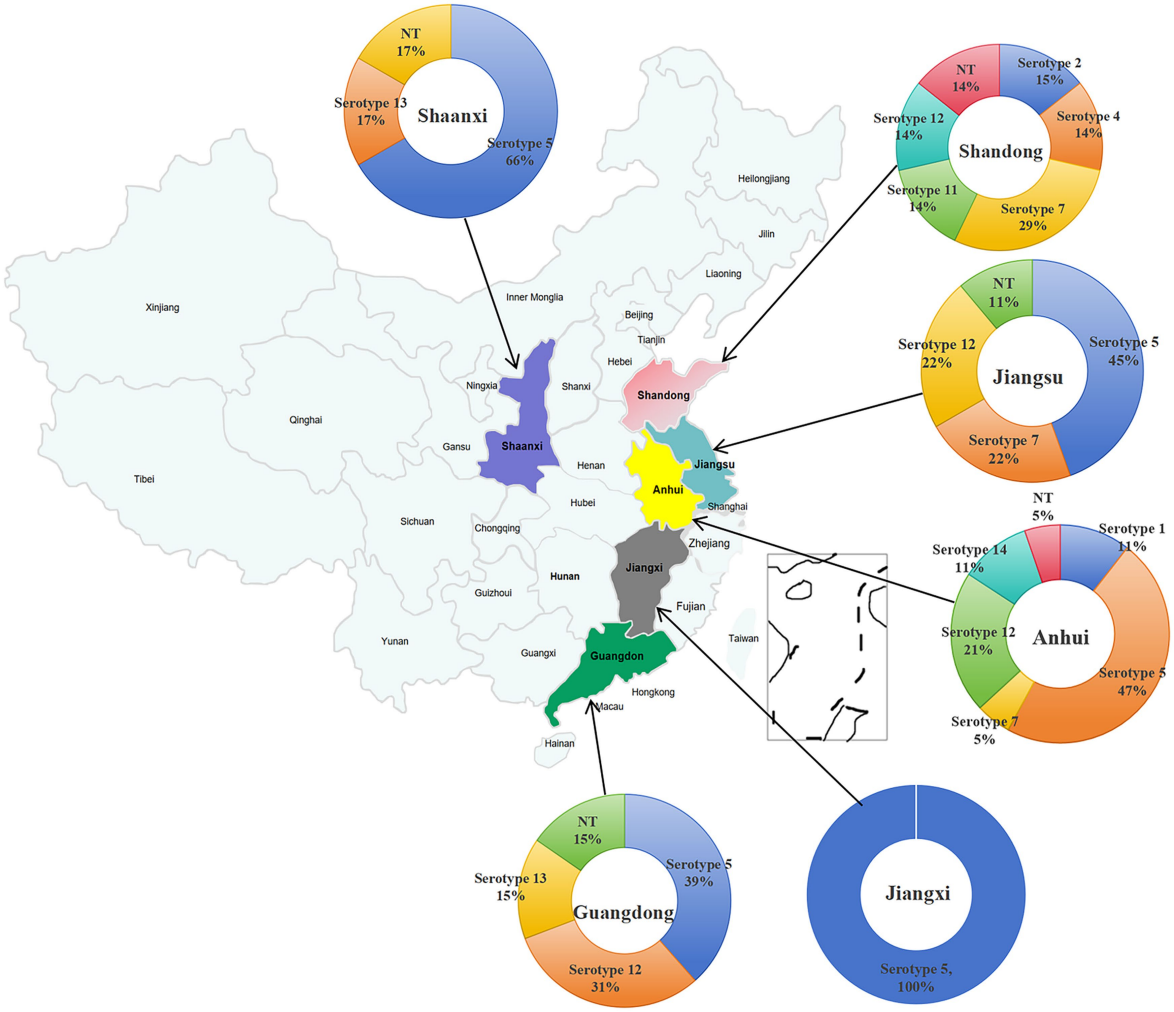
Distribution characteristics of serotypes of *H. parasuis* isolates in each of the six different regions of China.

Serotyping of 56 *H. parasuis* isolates by conventional PCR revealed distinct geographical patterns (Figure 5). Jiangxi Province exhibited a single-serotype profile, whereas other provinces displayed patterns of multiple-serotype cocirculation, with Anhui Province harboring six serotypes. Serotype 5 was absent in Shandong Province, and serotype 12 was not detected in Shaanxi and Jiangxi Provinces.

### 3.3 Virulence gene profiling of *Haemophilus parasuis* strains

**Figure 6:**
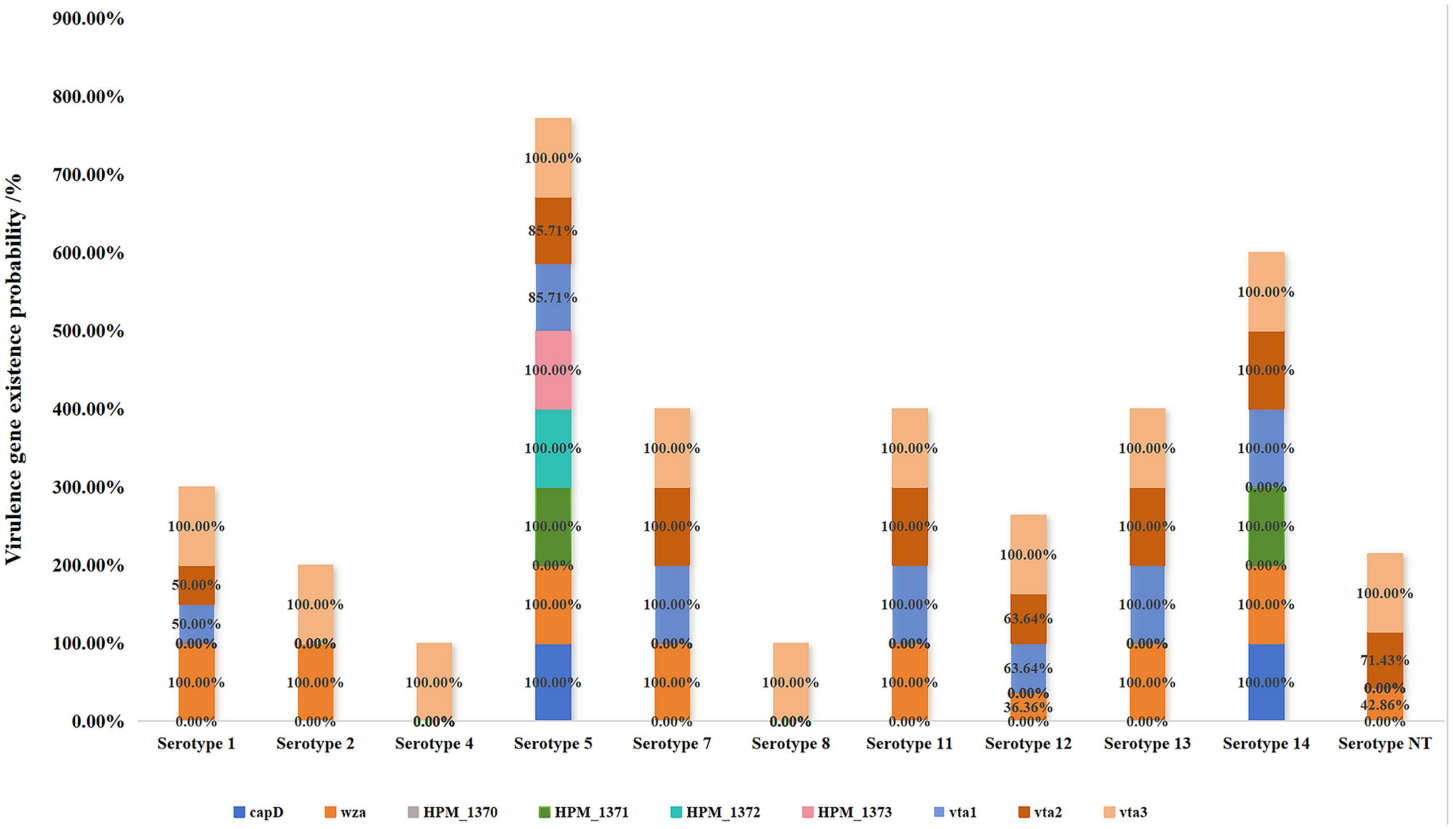
Association between the serotypes and virulence genes of 56 *H. parasuis* isolates.

This study targeted 10 virulence-associated genes (*lsgB*, *capD*, *wza*, *HPM-1370*, *HPM-1371*, *HPM-1372*, *HPM-1373*, *vta1*, *vta2*, and *vta3*) for comprehensive analysis (Figure 6). The findings revealed a complete absence of *HPM-1370* across all 56 *H. parasuis* strains, in contrast to the ubiquitous presence of *vta3*, which exhibited a 100% detection rate. Notably, serotypes 4 and 8 exclusively retained *vta3* as their sole virulence determinant, whereas serotype 5 displayed the most extensive virulence repertoire. Despite lacking HPM-1370, serotype 5 demonstrated complete conservation of lsgB and HPM-1373, along with an 83.33% co-occurrence rate (20/24) of vta1 and vta2. The HPM-1371 gene exhibited strict serotype restriction, being uniquely detected in all serotype 5 (24/24) and serotype 14 (3/3) isolates. Although serotypes 1, 11, 12, and 13 shared the core *wza*+*vta1* + *vta2* + *vta3* gene cluster, serotype 12 exhibited notable intraserotypic divergence; only 36.36% (4/11) retained wza, whereas 63.64% (7/11) simultaneously expressed both vta1 and vta2. Serotypes 2 and 7 presented identical virulence signatures, strictly preserving the *wza*+*vta3* genomic configuration. These serotype-dependent virulence gene architectures provide essential molecular epidemiological evidence for understanding the pathogenicity determinants of *H. parasuis*.

### 3.4 Animal pathogenicity assay

To accurately evaluate the pathogenicity of the dominant *Haemophilus parasuis* strains isolated in this study (H5-1 [serotype 5], H7-1 [serotype 7], and H12-1 [serotype 12]), 42-day-old healthy Landrace pigs with uniform body size were selected for an animal pathogenicity experiment. These pigs were confirmed to be negative for both antigens and antibodies against *Haemophilus parasuis* and other exogenous pathogens, including CSF, ASF, PRRS, and PCV.

**Figure 7:**
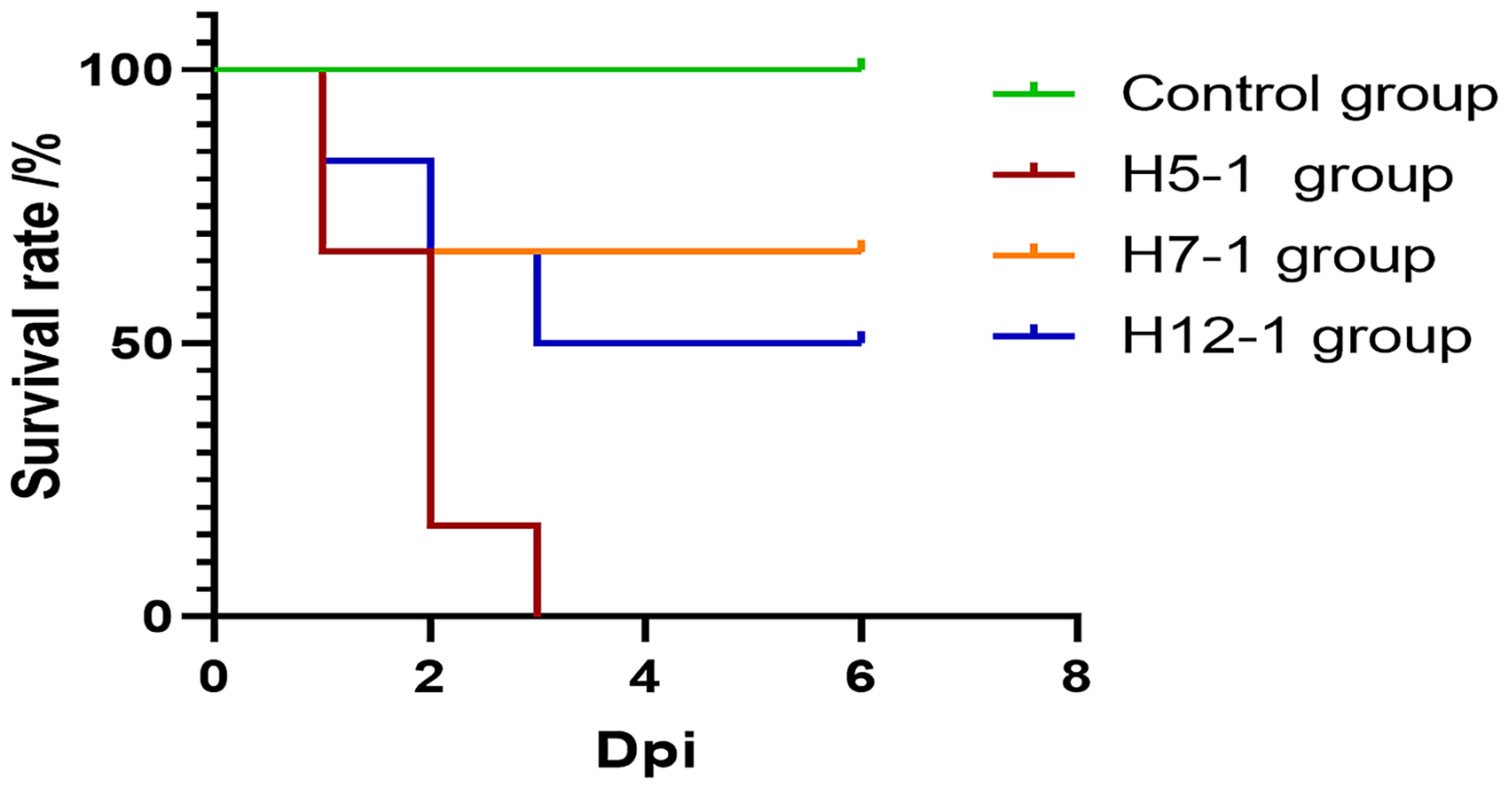
Survival curves of pigs after experimental infection. The mortality rates of the H5-1, H7-1, and H12-1 challenge groups were 100, 33.33, and 50%, respectively, whereas no deaths occurred in the control group.

During the experiment, the animals were divided into challenge groups and a control group. As shown in Figure 7, pigs in all three challenge groups exhibited typical symptoms such as lethargy, loss of appetite or anorexia, weight loss, and rough-textured hair coats following the challenge.

Notably, symptoms in the H5-1 and H12-1 challenge groups were particularly severe. In contrast, no overt lesions or mortalities were observed in the control group.

In the H5-1 challenge group, all six pigs exhibited symptoms of vomiting and abdominal breathing on the day of the challenge. Subsequently, these pigs succumbed one after another, resulting in a mortality rate of 100%. In the H7-1 challenge group, two pigs died acutely on the second day following the challenge. In contrast, the surviving pigs gradually recovered from their initial loss of appetite and returned to a normal state, resulting in a mortality rate of 33.33%. In the H12-1 challenge group, three pigs exhibited symptoms of vomiting and abdominal breathing on the second day after the challenge and subsequently died, resulting in a mortality rate of 50% (Figure 7).

**Figure 8:**
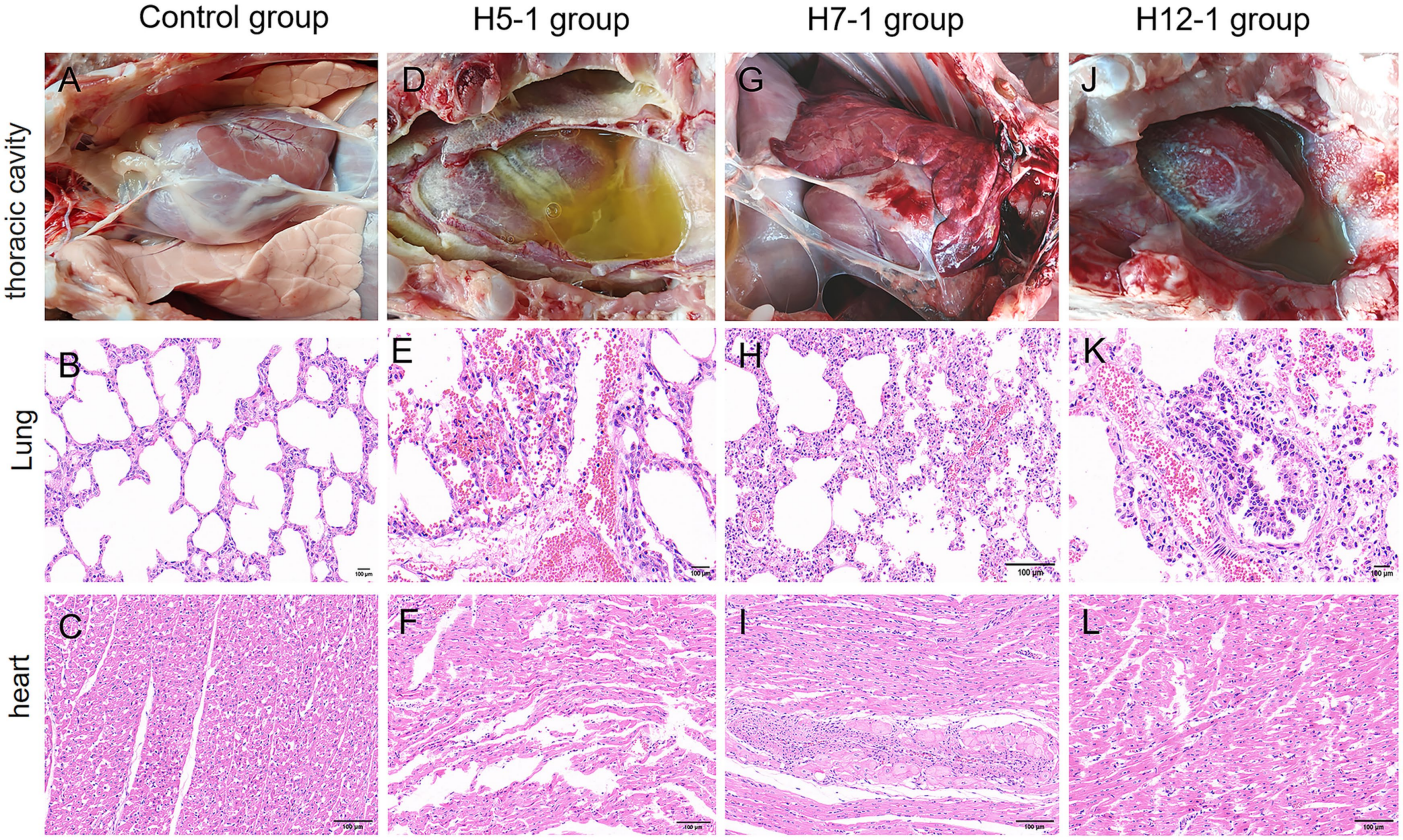
The degree of damage caused to organs in the thoracic cavity of pigs after experimental infection with f *H. parasuis*. **(A–C)** Gross changes in the thoracic cavity and histopathological changes in the lungs and heart of pigs in the blank group. **(D–F)** Gross changes in the thoracic cavity and histopathological changes in the lungs and heart of the pigs in the H5-1 challenge group. **(G–I)** Gross changes in the thoracic cavity and histopathological changes in the lungs and heart of the pigs in the H7-1 challenge group. **(J,K)** Gross changes in the thoracic cavity and histopathological changes in the lungs and heart of the pigs in the H12-1 challenge group.

To further investigate the extent of organ damage caused by the prevalent strains (serotypes 5, 7, and 12) in 42-day-old pigs, necropsy analyses were performed on the pigs that succumbed following the challenge. As illustrated in Figure 8D, the necropsy findings showed that in the H5-1 challenge group, a substantial accumulation of yellow fluid was observed in the thoracic cavity, accompanied by the exudation of white fibrin, resulting in severe adhesions between tissues. Additionally, signs of septicemia with impaired blood coagulation were evident. Histopathological examination revealed that the alveolar structure was ruptured or completely absent, with markedly widened interstitial edema, extensive hemorrhage, and infiltration of inflammatory cells (Figure 8E). The myocardial fibers were swollen or fractured, the striations appeared indistinct and disorganized, and prominent hemorrhage was observed in the capillary interstitium, accompanied by a significant increase in the number of inflammatory cells (Figure 8F). Necropsy of the pigs in the H7-1 challenge group revealed severe bleeding and congestion in the lungs. The lungs exhibited firmness upon palpation, and the pericardium of the heart was slightly enlarged; however, no other notable abnormalities were observed (Figure 8G). Histopathological analysis indicated that some alveoli were ruptured or absent, the interstitium was swollen and accompanied by hemorrhage, and the number of inflammatory cells was elevated (Figure 8H). The myocardial cells exhibited hypertrophy, accompanied by mild hemorrhage and infiltration of inflammatory cells (Figure 8I). In the H12-1 challenge group, necropsy revealed that a large volume of yellow fluid had accumulated in the thoracic cavity, which was accompanied by the exudation of white fibrin. Severe adhesions were observed among the lungs, heart, and pleura, along with extensive bleeding and congestion (Figure 8J). Pathology revealed that the alveolar structure in the pigs in this group had ruptured or had disappeared, with extensive bleeding in the interstitial region, accompanied by marked infiltration of inflammatory cells (Figure 8K). The myocardial fibers were fractured, the striations appeared disorganized, prominent hemorrhage was observed in the capillary interstitium, and there was a substantial increase in the number of inflammatory cells (Figure 8L). No evident lesions were detected in the control group (Figures 8A–C).

## 4 Discussion

*H. parasuis* is regarded as one of the most common bacterial infections affecting pigs worldwide. With the rapid development of the intensive farming model, the number of infection cases has increased significantly, further aggravating the severity of the antibiotic resistance problem (Cao et al., 2018; Zhao et al., 2024). Although previous studies reported an incidence rate of 27.8–41% in pig farms (Ni et al., 2020), the present study found that the overall infection rate across six provinces in China reached 52.10% (422/810), representing a substantial increase compared with historical data. Moreover, the infection rates during winter and spring were significantly higher than those in summer and autumn, consistent with previous findings and confirming the seasonal epidemic pattern of this pathogen. Notably, since the first report of *H. parasuis* in China in 1980, its prevalence has exhibited significant regional variation (Wang, 1980). For example, reported infection rates in Xinjiang (60.3%) (Li et al., 2014), Xiamen (72.0%) (Yang, 2014), Hainan (21.3%) (Wu et al., 2014), and Henan (21.0%) (Ma, 2017) vary markedly. The present study further confirmed this pattern, with Jiangxi Province exhibiting the highest infection rate at 71.76% (61/85), whereas Guangdong Province showed a relatively lower infection rate of 35.63% (31/87). This regional epidemic characteristic may be closely associated with factors such as climatic conditions, farming density, and the implementation of prevention and control measures.

Serotype 5 demonstrates a significant global epidemic trend. However, this study reveals that its epidemic characteristics differ markedly across various regions. Specifically, when compared to the epidemic levels in North America and Europe, the overall detection rate of serotype 5 in six provinces of China is notably higher, exhibiting a more pronounced regional characteristic (Macedo et al., 2021; Schuwerk et al., 2020). In addition, in this study, except for serotype 5, which was not detected in Shandong Province, it appeared in all other inspected areas. This finding indicates that Shandong Province may have formed a herd immunity barrier through vaccination strategies or natural exposure, thus inhibiting the prevalence of serotype 5. However, serotype 5 widely appears in other provinces with high infection rates, which also indicates that establishing a regional monitoring system and developing effective vaccines to address the impact of this serotype on pig farms are urgently needed.

The *lsgB* is expressed only in pathogenic *H. parasuis*, and it can significantly increase the pathogenic potential of a strain by increasing its serum resistance and immune escape ability (Martinez-Moliner et al., 2012). This study revealed that serotype 5, as the only serotype carrying the *lsgB* gene, has a 100% fatality rate. However, although *lsgB* was not detected in highly pathogenic serotype 12 or non-pathogenic serotype 7, there were also deaths of pigs and lesions in the thoracic organs. These findings indicate that *lsgB* may be a key factor for the high pathogenic virulence of serotype 5. However, other serotypes can cause diseases through alternative virulence factors or immune escape mechanisms.

Four glycosyltransferases (*HPM*-*1370*, *HPM*-*1371*, *HPM*-*1372*, *HPM*-*1373*) play key regulatory roles in the synthesis of bacterial surface molecules, and their presence or absence is associated with the immunogenicity of pathogenic bacteria and the ability of bacteria to evade phagocytosis (Lawrence et al.,2015). This study showed that only serotype 5 simultaneously carried the combination of *HPM-1370*, *HPM-1371*, HPM-*1372*, and *HPM*-*1373*. This finding is inconsistent with previous reports indicating that *H. parasuis* serotype 5 lacks the polysaccharide export protein, which may be attributed to the functional overlap of ABC transporters involved in polysaccharide polymer export (Lawrence et al., 2015).

Previous studies have demonstrated that *vta3* is highly conserved in both pathogenic and non-pathogenic strains, whereas *vta1* and *vta2* are primarily identified in pathogenic strains (Pina et al., 2009; Wu et al., 2023). Furthermore, some researchers have reported that non-pathogenic strains are positive for all three *vta* genes, while highly, moderately, and mildly pathogenic strains are positive only for *vta1* and *vta3*. In this study, all *H. parasuis* isolates carried *vta3*. *vta1* and *vta2* were also detected in mildly pathogenic serotypes (serotypes 7 and 11) and highly pathogenic serotypes (serotypes 5, 11, 12, and 13) (Olvera et al., 2012); however, these genes were not found in moderately pathogenic strains (serotype 4). This phenomenon may be attributed to the limited sample size of serotype 4 isolates, gene silencing, or functional redundancy under host selection pressure.

The *capD* gene encodes a polysaccharide biosynthesis protein associated with the pathogenicity of *Streptococcus suis* and is frequently detected in strains with moderate to high pathogenicity (Zhou et al., 2012). This study identified *capD* exclusively in highly pathogenic strains (serotypes 5 and 14), suggesting that *capD* could serve as a potential molecular marker for highly pathogenic strains (Wang et al., 2013). However, its absence did not appear to completely impair the adaptability of moderately pathogenic strains (Howell et al., 2013). The polysaccharide export protein *wza* has been reported to be highly conserved in the *H. parasuis* genome, which is consistent with the findings of this study (Reid and Whitfield, 2005).

*H. parasuis* is the causative agent of Glässer’s disease in pigs, and its infection often results in systemic diseases such as polyserositis, polyarthritis, and meningitis (Oliveira and Pijoan, 2004; Cai et al., 2005; Dickerman et al., 2020). According to the current classification system, *H. parasuis* is divided into 15 standard serotypes, with significant differences in pathogenicity observed among the different seroty (Zhou et al., 2016). Traditionally, serotypes 1, 5, 10, 12, 13, and 14 are considered highly pathogenic and can cause high mortality; serotypes 2, 4, 8, and 15 have lower pathogenicity; and serotypes 3, 6, 7, 9, and 11 are classified as non-pathogenic (Rapp-Gabrielson and Gabrielson, 1992). In the study, serotypes 5 and 12 caused a mortality rate of 50–100% in 42-day-old experimental pigs, which is consistent with reports classifying them as highly pathogenic serotypes. However, although serotype 7 is considered non-pathogenic, it caused 33% of the experimental pigs to die and induced mild lesions in the thoracic organs. This phenomenon may be related to the fact that the H7-1 isolate fully carries the vta1-vta2-vta3 virulence gene cluster, with a detection rate of 100%. This suggests that the isolate may have acquired pathogenicity islands from other highly pathogenic serotypes through horizontal gene transfer events, thereby disrupting the conventional association between serotype and pathogenicity (Oliveira and Pijoan, 2004).

Overall, this study systematically analyzed the epidemiological characteristics of *H. parasuis* in six provinces of China from 2022 to 2024. The results revealed that the national infection rate was significantly higher than previously reported, with a distinct seasonal trend characterized by higher infection rates during winter and spring. Serotypes 5 and 12 remained the predominant types, and the observed diversity in virulence gene combinations indicated variations in pathogenic mechanisms. Serotypes 5, 12, and 7 were all capable of causing clinical symptoms resembling those associated with *H. parasuis* disease, with serotype 5 exhibiting significantly stronger pathogenicity compared to serotypes 7 and 12. This study provides essential scientific evidence for the development of precise prevention and control strategies.

## Data Availability

The original contributions presented in the study are included in the article/Supplementary material, further inquiries can be directed to the corresponding authors.
